# Supplementary material for: Ferroelectric compute-in-memory annealer for combinatorial optimization problems
Source: Nat Commun. 2024 Mar 18;15:2419. doi: 10.1038/s41467-024-46640-x (PMC10948773; doi:10.1038/s41467-024-46640-x)
Supplement: Supplementary file 1 — Supplementary Information [file 41467_2024_46640_MOESM1_ESM.pdf]

Supplementary Information

1 Conversions from COPs to QUBO formulation

1.1 Max-Cut

Max-Cut is a well-known optimization problem in computer science and graph theory. Given an undirected graph $G(V, E)$ composing of a vertex set V and an edge set E, the object is to partition its vertices into two sets such that the number of edges crossing the partition is maximized [12].

To convert a Max-Cut problem into the Quadratic Unconstrained Binary Optimization (QUBO) formulation, we define a binary variable $x_i \in \{0, 1\}$ for each vertex i in the graph, such that $x_i = 1$ if vertex i is assigned to one set and $x_i = 0$ if it is assigned to the other set. The Max-Cut objective function can then be expressed as

$$\max \sum_{(i,j)\in E} (x_i + x_j - 2x_i x_j) \tag{4}$$

where E is the set of edges in the graph. The term $(x_i + x_j - x_i x_j)$ takes the value 1 when the adjacent vertices i and j are assigned to different sets, and 0 otherwise. Without loss of generality, for all the edges (i, j) in the graph, the object function can be rewritten as

$$\min \sum_{(i,j)\in E} (2x_i x_j - x_i - x_j) \tag{5}$$

which is in form of

$$\min y = \mathbf{x}^T Q \mathbf{x} \tag{6}$$

1.2 Graph Coloring

The graph coloring problem is described as follows. Given an undirected graph $G = (V, E)$, where V is the set of vertices and E is the set of edges, the object is to assign a color

to each vertex such that each pair of adjacent vertices have different colors. That is, to find a desired vertex coloring function of a graph G $f : V \rightarrow C$ where C is a set of colors, such that $f(u) \neq f(v)$ for every pair of adjacent vertices u and v in G. Specifically, the K-coloring problem whose object is to find a vertex coloring of a graph using K colors, is a classic problem in graph theory and has been extensively studied for a variety of applications including frequency assignment problems and printed circuit board design problems [12].

To convert a K-graph coloring problem into the QUBO formulation, we define a binary variable $x_{ip} \in \{0, 1\}$ for a node coloring, such that $x_{ip} = 1$ if node i is assigned color p, and 0 otherwise. Since each node must be colored by one color, we have the constraint

$$\sum_{p=1}^{K} x_{ip} = 1 \quad i = 1, ..., n \tag{7}$$

where n denotes the number of nodes in the graph. All the edges (m, n) in the graph are required to connect different colors, leading to the constraint

$$x_{mp} + x_{np} \leq 1 \quad p = 1, ..., K \tag{8}$$

The corresponding quadratic penalty for the constraint Eq. 8 is assumed as

$$x_{mp} x_{np} \quad p = 1, ..., K \tag{9}$$

The penalty is not added only when the colors of adjacent nodes are different. The object function of graph coloring problem can then be expressed as a QUBO formulation:

$$\min y = \sum_{i \in V} \sum_{p}^{K} (x_{ip} - 1)^2 + \sum_{(m,n) \in E} \sum_{p}^{K} x_{mp} x_{np} \tag{10}$$

1.3 Prime Factorization Problem

The prime factorization problem (PFP) aims to find the prime factors of a large integer. Fig. S1 conceptually illustrates an example of converting to QUBO formulation. Suppose we have an integer $N = P \times Q$ that needs to be factored, P and Q are firstly rewritten as $P = (1 p_k p_{k-1} ... p_1 1)_2$ and $Q = (1 q_l q_{l-1} ... q_1 1)_2$, respectively. The multiplication of $P \times Q$

is expanded as bit-wise partial products listed in the multiplication table. Then the bit-wise partial products of the two factors are grouped as integer blocks and sum to the corresponding integer blocks of the given integer N, forming the integer block equations. By introducing auxiliary variables, the objective function derived from the squared error of block equations is reduced to QUBO formulation.

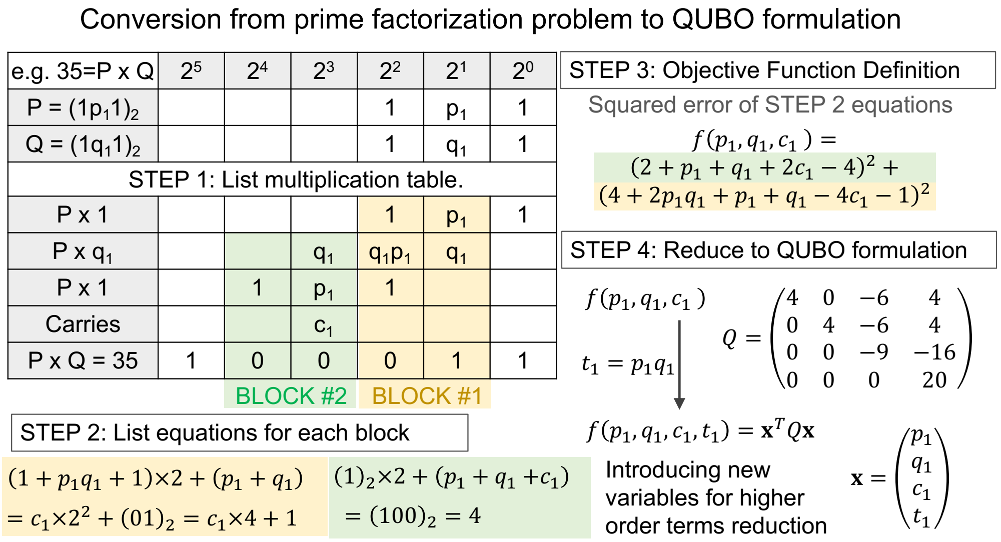

Figure S1: Conceptual conversion from prime factorization to $\mathbf{x}^T Q \mathbf{x}$ QUBO formulation by leveraging the block-wise binary multiplication table, objective function formulation and higher order term reduction methods.

2 Flow of proposed framework

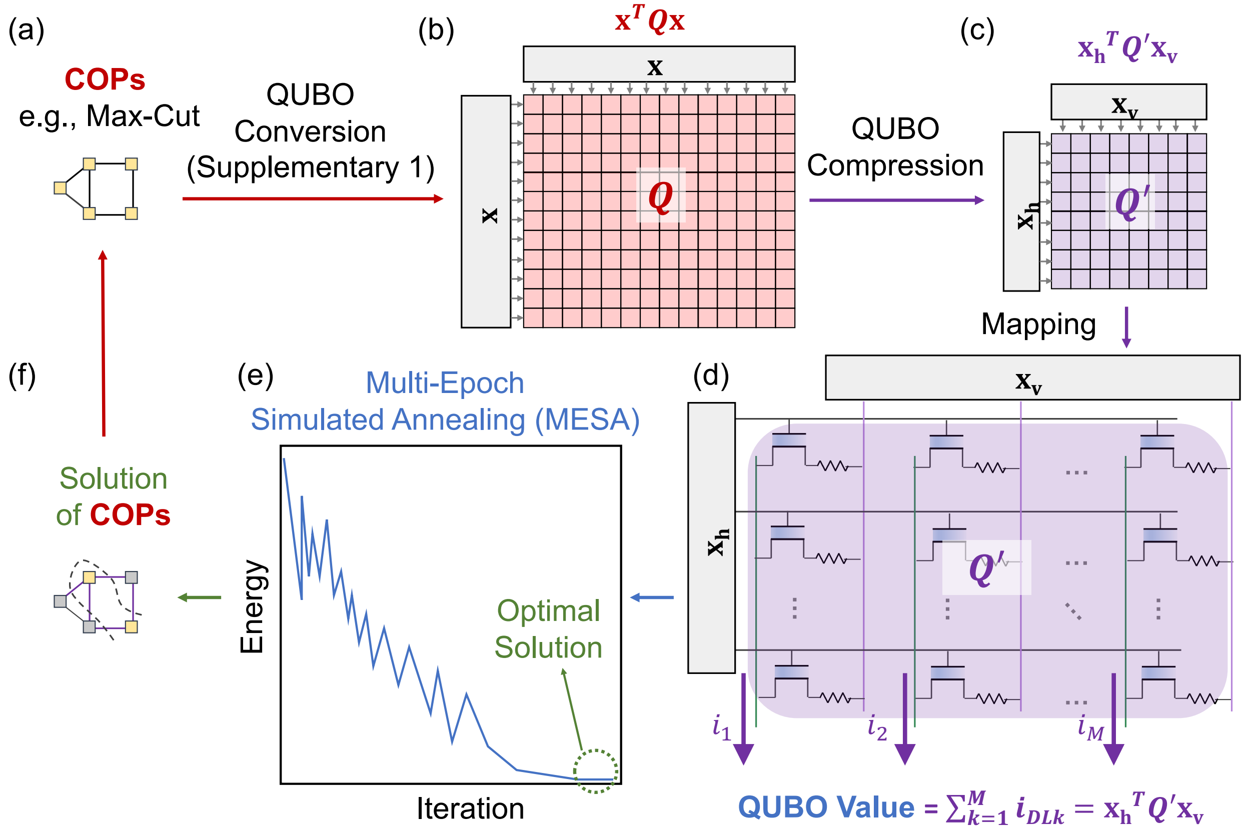

Figure S2: **Flow of our proposed framework.** **a.** Various types of COPs are converted to **b.** QUBO form $\mathbf{x}^T Q \mathbf{x}$, which is **c.** losslessly compressed into more general and compact $\mathbf{x_h}^T Q' \mathbf{x_v}$. **d.** $\mathbf{x_h}^T Q' \mathbf{x_v}$ is mapped to FeFET-based crossbar for QUBO computation, **e.** within each iteration of MESA process. **f.** The solutions of the COPs are ultimately obtained.

Fig.S2 depicts the flow of our proposed framework, and we illustrate its working principle as below:

STEP 1: A COP is converted into a QUBO formulation $\mathbf{x}^T Q \mathbf{x}$.

STEP 2: This QUBO formulation is losslessly compressed into a more general and

compact form with asymmetric variable mathbftors $\mathbf{x_h}^T Q' \mathbf{x_v}$.

STEP 3: The QUBO matrix Q' of the compressed formulation is mapped onto a FeFET-based crossbar array, which inherently performs single-step VMV multiplication. The summed current of the crossbar represents the value of the compressed QUBO formulation.

STEP 4: The solving process utilizes a multi-epoch simulated annealing (MESA) algorithm. In each iteration, the FeFET-based array computes the QUBO formulation value, and the objective function value is determined.

STEP 5: After the MESA process, the variable mathbftor configurations that correspond to the optimal objective function value are obtained and translated into the solution for the given COP.

3 FeFET CiM chip integration & testing

The electrical characterization setup is shown in Fig. S3**a**. The measurements are performed mainly with a PXIe measurement system from National Instruments (i.e., label 1 in Fig. S3). A padring of 28 individual analog and digital pads connect the 1kb (32×32) FeFET macro with a serial peripheral interface (SPI). The adapter board (label 2 in Fig.S3) connects to the specific pads on the wafer in a wafer probe system (label 3 in Fig. S3) via a probe-card (label 4 in Fig. S3), see zoomed in for Fig. S3**b**/**c**.

A set of separate NI PXIe-4143 source measure units (SMU) and an NI PXIe-6570 pattern generator are employed. Notably, the output pins of the latter device can be utilized as a Pin Parametric Measurement Unit (PPMU). Through this arrangement, the requisite supply, bias voltages, and digital signals are generated. Additionally, the pattern generator plays an instrumental role in forming the scan chain for the appropriate addressing of wordlines and sourceline/drainline.

For the task of loading mathbftors x and y, an external clock, operating at an upper limit of 50 MHz, is employed. The wordline voltages designated for reading are sourced from the PPMU, covering both logical states '0' and '1'. In the context of this experiment, these states correspond to 0V and 1.2V, respectively, during the read phase. For this experimental demonstration, we posit a ternary precision for the Q matrix. This matrix is depicted through the decomposition of ternary weights into a 2FeFET cell. This arrangement obviates the need for an ADC and shift-add operations. Consequently, all binary operations can be aggregated across the entire matrix within a singular cell, thereby streamlining and accelerating the overarching operation. The macro is equipped to support both single-bit reading and parallel decoders, ensuring maximal flexibility in wordline/bitline combinations. The matrix also features adaptable bitline multiplexers, facilitating the selection of the active bitlines. In culmination, the current across all active bitlines y in the 1kb 1FeFET-1R array, and by extension the xQy outcome, is ascertained in a singular read cycle with a designated bitline voltage of 0.1V.

The matrix parameters of Q are programmed word by word into the FeFET array following a $V_W/3$ inhibit scheme (V_W is the write voltage). Specifically, we apply a pulse of 3.3V for the selected wordlines, 0.8V to unselected wordlines as well as 0V to selected and 1.8V to non-selected sourcelines with a duration of 10μs. More programming dynamics and parameters and chip specifications are illustrated in Fig. S4 and S5.

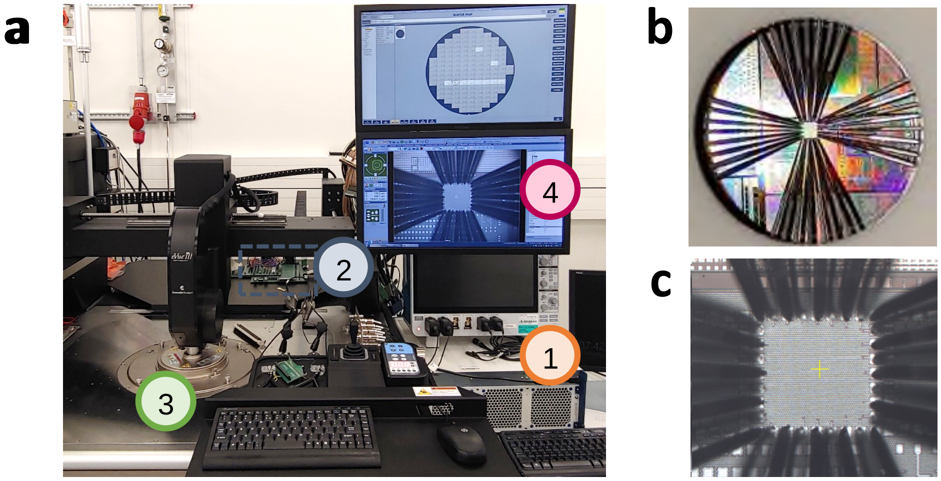

Figure S3: **a.** Experimental setup for FeFET characterization. A PXI System is employed, providing both Source Measurement Units (SMU) and Pin Parametric Measurement Units (PPMU). PPMUs facilitate the configuration of the Switch Matrix for directing the source signals to the respective contact needles. **b.** Test structures, situated on on 300 mm wafers, are interfaced with the measurement setup through a semi-automatic probe station, using **c.** a probe card for connection.

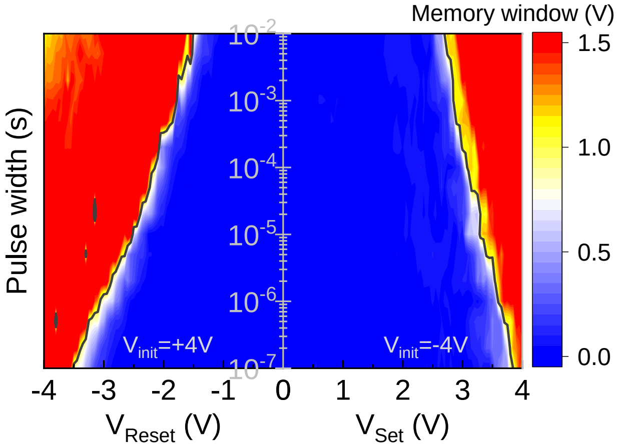

Figure S4: ***Switching dynamics of a FeFET under different write pulse amplitudes and pulse width.*** *The color represents the memory window. The boundary line indicates a memory window of 1V. The left half figure represents the scenario that a FeFET is initialized to 4V every time and then a negative reset gate pulse is applied, following which the memory state is measured. Similarly for the right half figure, the device is initialized with -4V write pulse.*

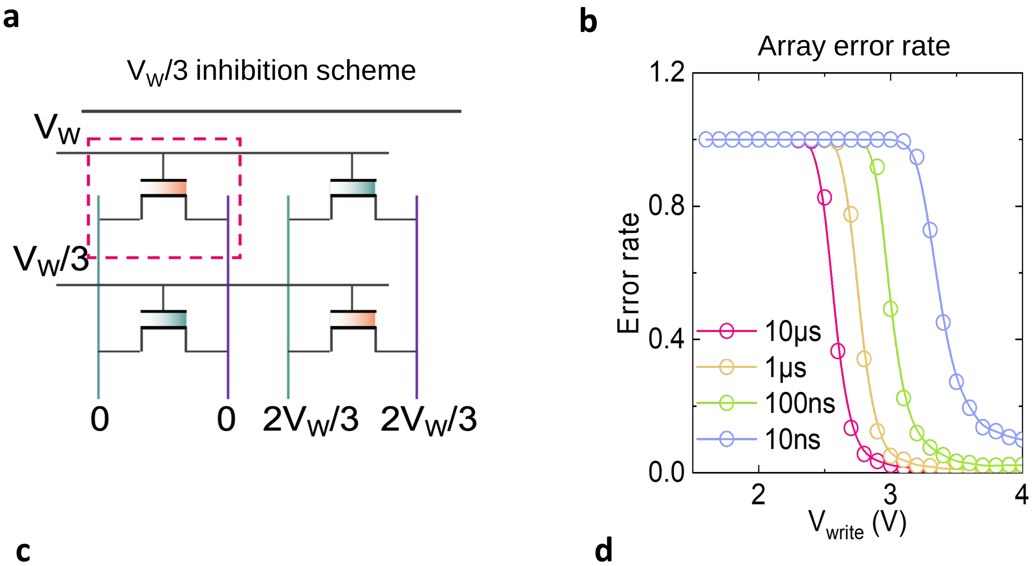

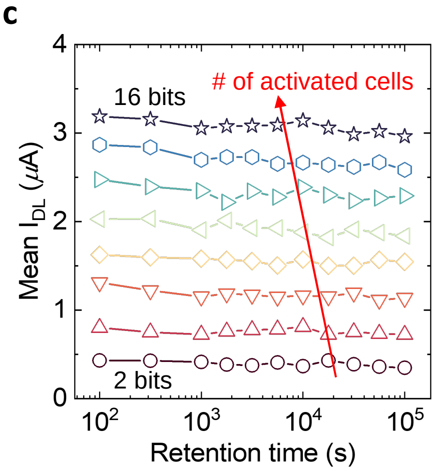

d

Technology	28nm CMOS Logic Process
FeFET technology	FEOL FeFET
Cell Type	1FeFET1R
FeFET dimension	0.45µm x 0.45µm (WxL)
Array size	32 rows x 32 columns
Read Voltage	1.2V
Program Voltage	3.3V
VDD	1V
VDDIO	3.3V

Figure S5: Programming conditions of the integrated FeFET array. **a**, Configuration of parallel program following wordline and bitline/source inhibit for passive program. **b**, Investigation of programming condition of the kb array in dependence of program pulse voltage and duration; **c**, retention performance of the array at room temperature while performing the compute-in-memory operation; **d**, chip specifications.

4 Lossless QUBO compression

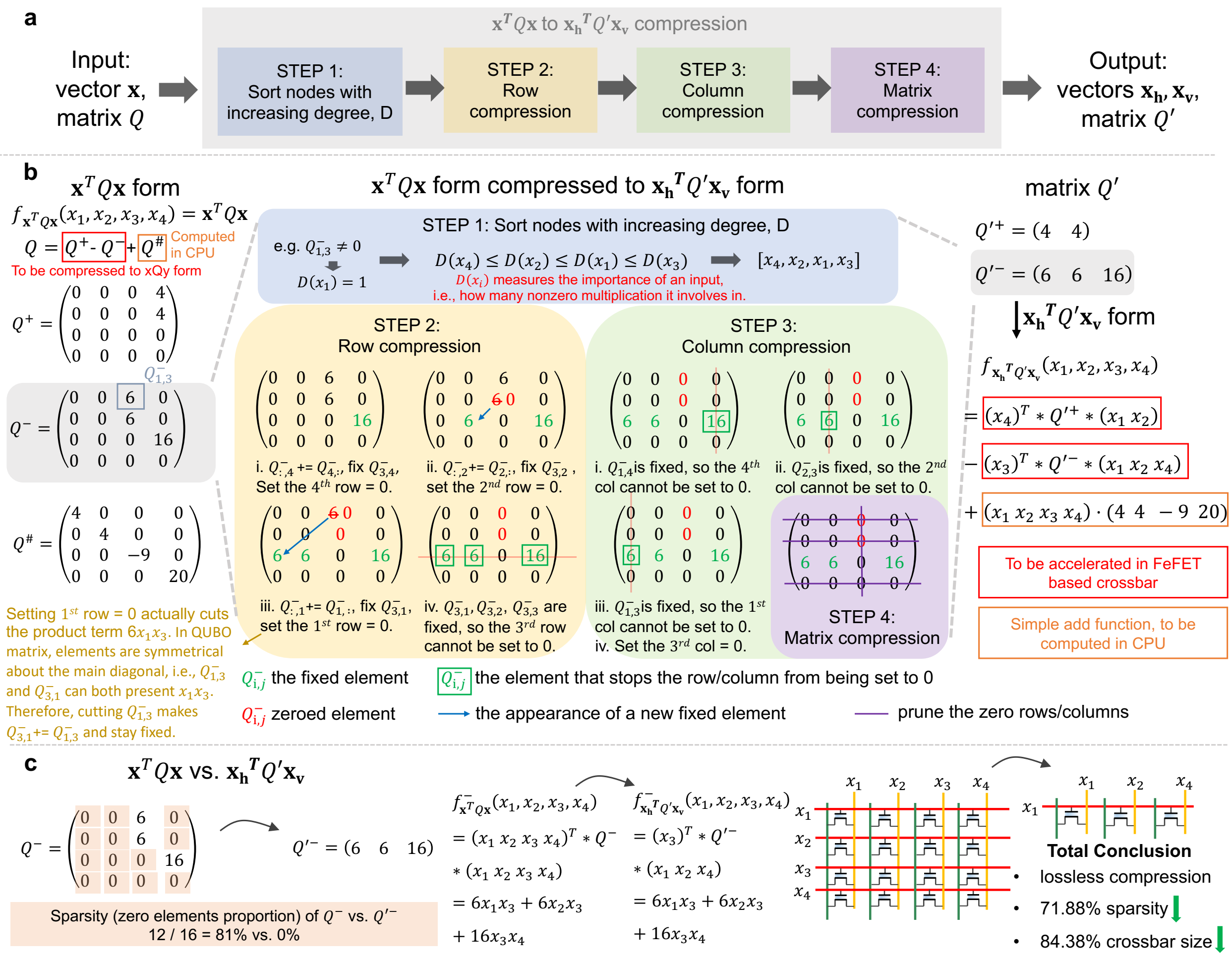

Figure S6: **a**, Flow of QUBO compression from $\mathbf{x}^T Q\mathbf{x}$ to $\mathbf{x_h}^T Q'\mathbf{x_v}$ QUBO formulation. **b**, Compression example of the PFP 35=5×7 QUBO matrix; **c**, The significant savings of the lossless QUBO compression in **b**, respectively.

Fig. S6a and b illustrate the QUBO compression flow and a detailed example of the PFP converted QUBO compression, respectively. Following the compression steps described in the main text, the QUBO matrixes converted from the PFP 35=5×7 are compressed as below (take Q^- as the example):

STEP 1: All the input variables $\mathbf{x}$ are sorted in a connectivity increasing order, i.e., CIA=$[x4, x2, x1, x3]$.

STEP 2: Row compression of Q matrix is performed in the order of CIA. For variable x_4 selected from CIA, its corresponding 4th row contains no *fixed* element, thus this row can be compressed, denoted as compressed row. All the elements $Q^-_{4,j}, j \in [1,4]$ within the row are added to their diagonal elements, i.e., $Q^-_{j,4} + = Q^-_{4,j}, j \in [1,4]$. The diagonal element $Q^-_{3,4}$ is nonzero, thus *fixed* to ensure the presence of the associated product term, i.e., $16x_3x_4$ in QUBO formulation. For variable x_2, the corresponding 2nd row contains no *fixed* element, this row can be compressed, denoted as compressed row. The elements $Q^-_{2,j}, j \in [1,4]$ are added to their diagonal element $Q^-_{j,2}$, set to 0, and then their diagonal nonzero element $Q^-_{3,2}$ is *fixed* to keep $6x_3x_2$ in QUBO formulation. For variable x_1, its corresponding row contains no *fixed* element, thus can be compressed. The elements $Q^-_{1,j}, j \in [1,4]$ are added to the diagonal elements $Q^-_{j,1}$, set to 0 and the diagonal nonzero element $Q^-_{3,1}$ is *fixed* to keep $6x_3x_1$ in QUBO formulation. For the last variable x_3, its corresponding row contains *fixed* elements $Q^-_{3,1}$, $Q^-_{3,2}$ and $Q^-_{3,4}$, thus cannot be compressed.

STEP 3: Column compression follows the similar operation to the row compression in Step 2. Following the CIA, the 4th, 2nd, 1st columns contain *fixed* elements $Q^-_{3,4}$, $Q^-_{3,2}$, $Q^-_{3,1}$, respectively, thus cannot be compressed. The 3rd column contains no *fixed* elements, thus denoted as compressed column. The elements within the column are zero, and their diagonal elements are zero, thus the 3rd column can be set to 0.

STEP 4: The compressed rows, i.e., the 1st, 2nd, 4th rows and the compressed column, i.e., the 3rd column of the QUBO matrix along with their respective variables in the input mathbftors, i.e., x_1, x_2, x_4 in $\mathbf{x_h}$ and x_3 in $\mathbf{x_v}$, are removed, forming the compressed QUBO formulation, i.e., $x_3^T Q^{'-}(x_1, x_2, x_4)$, $Q^{'-} = (6, 6, 16)$.

Fig. S6c shows the compressed QUBO formulation and its crossbar size used to map the matrix. The binary variable mathbftors $\mathbf{x_h}/\mathbf{x_v}$ are applied to the WLs/SLs of the FeFET crossbar array to implement iterative mathbftor-matrix-mathbftor multiplications. In this PFP example, two QUBO matrixes (Q^+ and Q^-) are compressed, achieving 71.88% sparsity reduction and 84.38% chip area saving compared to the original QUBO implementation.

5 Example: MESA analysis over Max-Cut problem

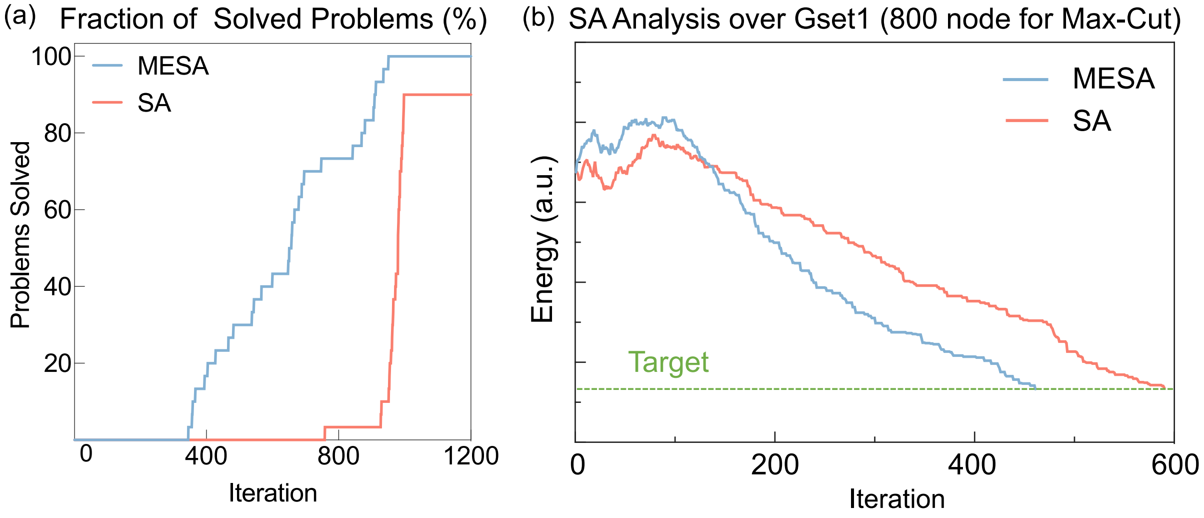

Figure S7: **a**, The fraction of solved problems and time-to-solution of MESA compared to conventional SA in solving Max-Cut problems. **b**, Energy evolution of MESA and SA in solving a Max-Cut problem with 800 nodes.

Fig. S7(a) depicts comparison between MESA and conventional SA in solving Max-Cut problems. Results show that the fraction of solved problems by MESA gradually grows with iteration count, and eventually reaches 100%. In contrast, the fraction of solved problems by conventional SA remains 0 until nearly 1,000 iterations, and ultimately reaches 90% with more iteration count. Fig. S7(b) illustrates the energy evolution of MESA and conventional SA in solving a Max-Cut problem with 800 nodes. Clearly, MESA achieves the target result within fewer iterations, whereas conventional SA fails to find the optimal solution within the same time frame.

6 Example: prime factorization problem (PFP) of 323

Fig. S8a shows the MESA process in searching for the solution of factoring 323 within one SA epoch, where the number of input variables is 10, and the QUBO coefficient precision is set as 5-bit. The corresponding energy evolution of the converted QUBO formulation is shown in Fig. S8b. Fig. S8c shows the success rates of solving the PFP of 323, which is defined as the probability of finding the optimal factors. The results suggest that insufficient weight precision stored in the compute-in-memory crossbar may lead to imprecise solutions, as the energy landscape with quantized QUBO formulation deviates significantly from the real energy landscape. High success rate can be achieved with 5-bit precision configuration. Fig. S8d shows that most of the minimal attractors found by our proposed framework with MES are validated to be optimal/correct solutions.

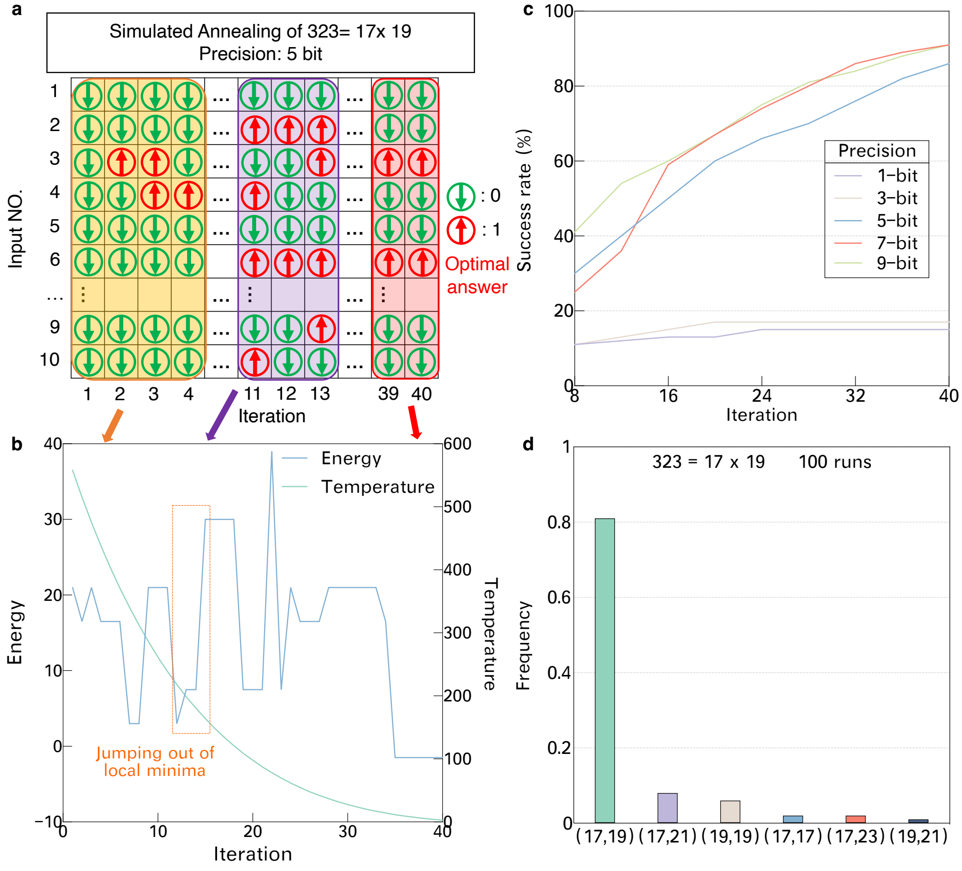

Figure S8: **a**, Exemplar MESA process and **b**, the corresponding energy evolution process

of PFP solving. **c**, The success rates of factoring 323 with different weight precisions. **d**, Attractor occurrences for factoring 323.

To achieve a higher success rate for solving PFPs, increasing the QUBO coefficient precision is an option, as illustrated in Fig. S9. That said, higher precision requires higher analog-digital-converter (ADC) resolution to accurately compute the QUBO formulation. Therefore, higher success rate comes at the cost of extra energy and hardware overheads.

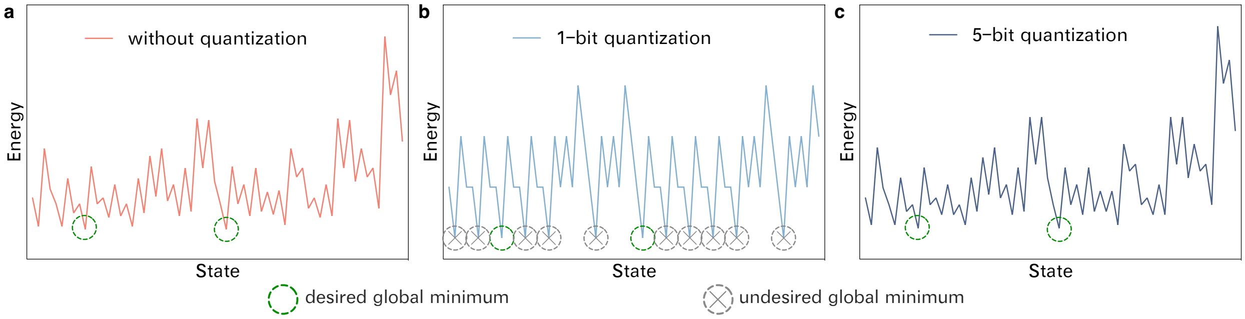

Figure S9: To **a**, The ideal energy landscape of QUBO formulation when solving 323 = 17×19. **b**, Low bit quantization of QUBO coefficient results in numerous erroneous global minima.**c**, The landscape under high bit quantization exhibits no erroneous global minima, thus enhancing the success rate, as illustrated in Fig. S8c.

7 Experimental demonstration of graph coloring problem

Fig. S10 demonstrates the QUBO energy functions measured on our fabricated FeFET crossbar prototypes for solving the exemplar graph coloring problem depicted in Fig. 4. As can be seen from the energy trajectories, all three prototypes are capable of finding the optimal solution of the problem within 100 iterations, thus suggesting the robustness of both our prototypes and the proposed hardware-algorithm co-design framework. Moreover, the intra-prototype variation of energy curves is negligible, highlighting the performance consistency of our fabricated hardware.

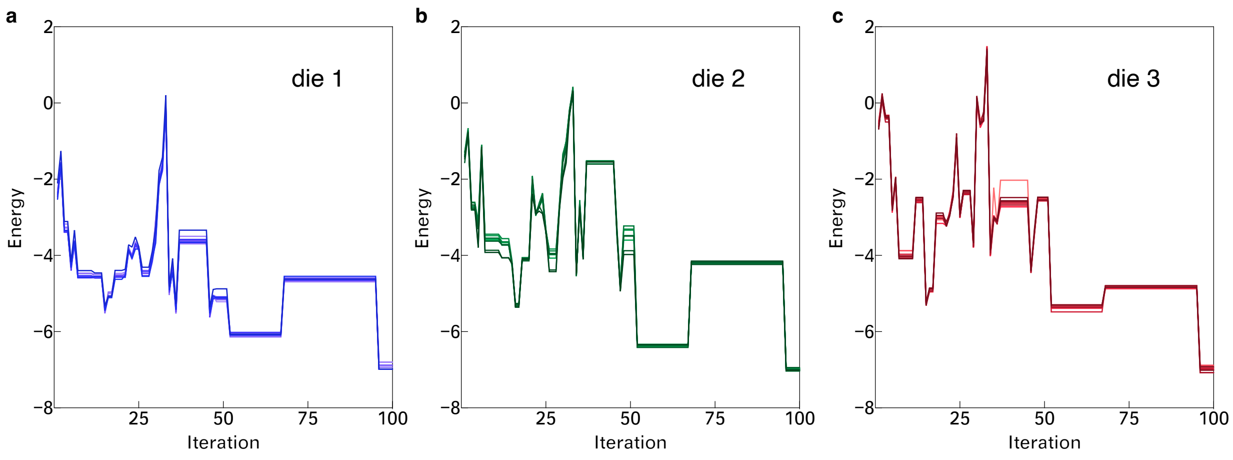

Figure S10: Experimental results for solving the graph coloring problem, as shown in Fig. 4f. The energy curves for three different dies **a**/**b**/**c**, with each die running the problem-solving process 9 times.

Fig. S11 showcases the graph coloring configurations during annealing, highlighted at different iteration steps shown in Fig.4*i*, i.e., the beginning (A), midpoint (B), and end (C) of the evolution process.

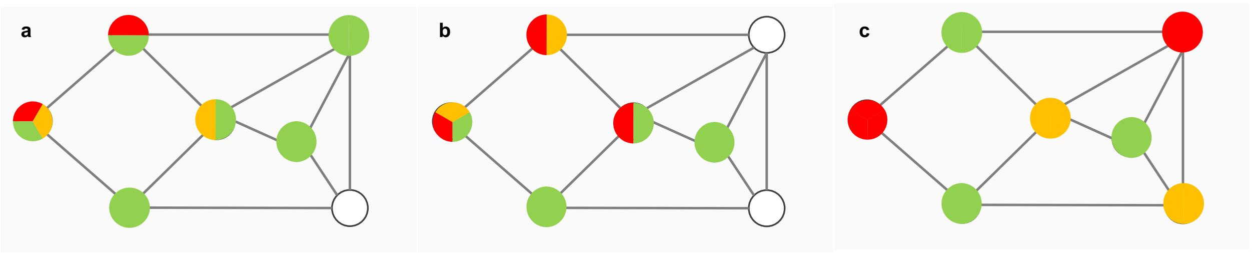

Figure S11: Graph coloring configurations at annealing stages (**a**, **b**, **c**) during the evolution process. White means no color assignment. Nodes with one, two, three colors means that one, two, or three colors have been assigned to the nodes. Note that more than one color does not meet the constraint and no longer exists at the final converged solution.

8 Addressing larger-scale COPs

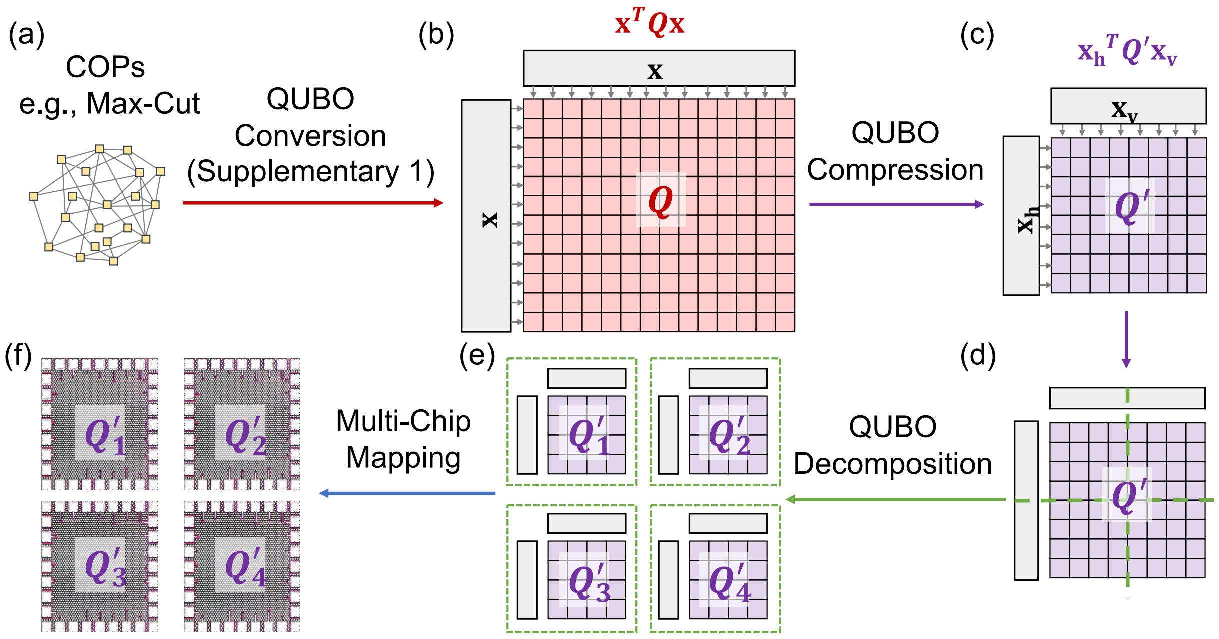

Figure S12: **Large-scale COPs solved by multi FeFET chips. a.** Large-scale COPs. **b.** COPs converted to general QUBO form $\mathbf{x}^T Q\mathbf{x}$. **c.** $\mathbf{x}^T Q\mathbf{x}$ losslessly compressed into $\mathbf{x_h}^T Q'\mathbf{x_v}$. **d.** Q' being decomposed into multiple sub-matrices. **e.** $\mathbf{x_h}^T Q'\mathbf{x_v}$ being decomposed into multiple forms. **f.** Multiple forms being mapped to multiple chips for $\mathbf{x_h}^T Q'\mathbf{x_v}$ computations.

Besides the experimental demonstration of graph coloring problem, Section 1 of Supplementary Information has thoroughly described how various COPs can be converted into QUBO forms. After conversions, our fabricated FeFET chip can address the converted QUBO formulation and identify the optimal variable values. These values can then be translated into the corresponding solutions for these COPs. That is, our proposed chip can fit with other COPs following the same procedure described above.

When addressing larger-scale COPs that surpass the capacity of our chip, we employ a decomposition and solution strategy, as depicted in Fig. S12. Initially, the large-scale COP (e.g., Max-Cut) (a) undergoes the conversion to QUBO form, followed by (b) a lossless compression into a (c) more compact QUBO form $\mathbf{x_h}^T Q'\mathbf{x_v}$. This condensed form (d)

is subsequently decomposed into several smaller forms (e), each of which is (f) mapped to individual FeFET chips for separate QUBO computations. The final QUBO value, corresponding to the original objective function of large-scale COP, results from combining the outputs of the decomposed forms processed on multiple chips. Or equivalently, these decomposed smaller forms are carried out in the time domain using a single FeFET chip. In this approach, the smaller matrices are sequentially programmed into the FeFET chip, and the calculated results are temporarily buffered and summed together at the end. By decomposing the corresponding QUBO formulation, calculating the decomposed QUBO forms across multiple chips or on a single chip, and aggregating the outputs of decomposed QUBO forms, this decomposition process showcases the efficiency of our proposed framework in managing larger-scale COPs. This strategy allows our chip to effectively handle larger-scale COPs while preserving good linearity and mitigating the impact of device non-ideality. Such scalability of our approach stands as a significant advantage compared to other Ising machines, particularly in addressing challenges related to scalability and problem decomposition for larger-scale COPs.
